# Selective hemoadsorption of cytokines and platelet-neutrophil complexes mitigates lung microvascular hyperpermeability in an ovine acute lung injury model

**DOI:** 10.1186/s40635-026-00888-3

**Published:** 2026-04-01

**Authors:** Satoshi Fukuda, Yosuke Niimi, Yumiko Sekiya, Ennert Manyeza, Sam Jacob, Robert A. Cox, Donald S. Prough, Hiroshi Takahashi, Keishi Miwa, Toru Kotani, Perenlei Enkhbaatar

**Affiliations:** 1 Department of Anesthesiology, University of Texas Medical Branch, Galveston, TX USA; 2 Department of General Internal Medicine, YUASA Foundation Jusendo General Hospital, Fukushima, Japan; 3 Department of Plastic and Reconstructive Surgery, Tokyo Women’s Medical University, Tokyo, Japan; 4 Medical Devices & Materials Research Lab., Advanced Materials Research Labs., Toray Industries, Inc., Shiga, Japan; 5 Department of Pathology, University of Texas Medical Branch, Galveston, TX USA; 6 Department of Intensive Care Medicine, Showa Medical University, Tokyo, Japan

**Keywords:** Platelet-neutrophil complex, Cytokine, Hemoadsorption column, Ovine, ARDS

## Abstract

**Background:**

Acute respiratory distress syndrome (ARDS) is a life-threatening disease that is characterized by noncardiogenic pulmonary edema, respiratory distress, and hypoxemia, and has high mortality. Uncontrolled activation of neutrophils and formation of platelet-neutrophil complexes (PNCs) contribute significantly to its pathogenesis. In this study, we investigated whether suppression of systemic inflammation through simultaneous adsorption of inflammatory mediators, including PNCs, reduces lung invasion and offers an effective treatment strategy for ARDS.

**Materials and methods:**

We investigated the efficacy of a novel hemoadsorption column, NOA-001, which simultaneously removes cytokines and PNCs, in an ovine model of smoke inhalation-induced ARDS. Animals were assigned to two groups: treatment with NOA-001 (n = 6) or a control group with a blood circuit without the column (n = 5). The impact on neutrophil dynamics, lung injury, edema formation, and inflammatory markers was assessed.

**Results:**

Neutrophil capture rates by NOA-001 were significantly higher at 4 and 6 h compared to controls, with a trend toward lower circulating neutrophil counts. Flow cytometry confirmed that NOA-001 predominantly removed PNCs (CD62^+^/CD11b^low^ or CD11b^high^). At 27 h, the NOA-001 group had significantly improved oxygenation (oxygenation index: 10.3 ± 3.0 vs. 18.8 ± 3.2, p < 0.05) and significantly lower lung injury scores (1.9 ± 0.3 vs. 2.8 ± 0.2, p < 0.05) compared to controls. Lung edema was significantly attenuated, as evidenced by reduced lung lymph flow (27.3 ± 6.4 vs. 57.8 ± 6.4 mL/h), lower wet-to-dry weight ratios in lung (7.4 ± 0.3 vs. 9.2 ± 0.6) and trachea (2.7 ± 0.1 vs. 3.0 ± 0.1), and decreased thoracic exudate volume (90 ± 69 vs. 620 ± 183 mL, p < 0.05). Pulmonary protein leakage and neutrophil migration indices were also significantly lower in the NOA-001 group. Interleukin-6 levels in bronchoalveolar lavage fluid tended to be lower in the NOA-001 group (p = 0.08). No adverse events were observed.

**Conclusions:**

Simultaneous removal of cytokines and PNCs using NOA-001 significantly reduced ARDS severity by attenuating pulmonary edema and improving pulmonary gas exchange. These results suggest that NOA-001 may be a novel and promising therapeutic strategy for ARDS.

## Background

Acute respiratory distress syndrome (ARDS) is a life-threatening pulmonary disease caused by a dysregulated immune response to various insults, including pneumonia, sepsis, gastric aspiration, acute pancreatitis, and other diseases [1]. The pathophysiological mechanisms underlying ARDS are complex; however, pulmonary edema resulting from microvascular hyperpermeability due to proinflammatory cytokines and dysregulated activated neutrophils is considered to be a hallmark of severe lung injury [2]. Thus, ARDS treatment must address mitigation of both endothelial and epithelial lung injury.

In ARDS, neutrophils interact with platelets and form platelet-neutrophil complexes (PNCs) [3], which play a critical role in disease progression. First, activated platelets satellite to neutrophils in a CD62P (P-selectin)-dependent manner, and independent of CD11b (integrin αM) expression. The PNCs have enhanced adhesion to the endothelium compared to neutrophils alone. With increased CD11b expression on neutrophils due to the interaction of CD62P with P-selectin glycoprotein ligand-1 (PSGL-1), PNCs shows increased a trans-migration activity across the endothelium and release cytotoxic mediators that lead to endothelial injury, resulting in leakage of protein-rich fluid and migration of immune cells across the endothelium [4, 5]. There are two coexisting subpopulations of PNCs with low (PNC/CD11b^low^) and high (PNC/CD11b^high^) CD11b expression. PNCs also include neutrophil extracellular traps (NETs), which are web-like structures composed of extracellular DNA, histones, and proteins from neutrophil granules secreted in response to viruses, bacteria, immune complexes, and cytokines, and may be correlated with the severity of ARDS [2, 6].

Most clinical trials of drugs and stem cell therapy for ARDS have failed to show sufficient efficacy [7]. This may be due to the complex, multifactorial nature of the disease, which a single-target approach may not fully address. We hypothesized that modulation of multiple factors (cytokines, PNCs, and activated neutrophils) may be more effective than a single-target therapy in preventing ARDS progression. To this end, we developed a novel extracorporeal hemoadsorption column (NOA-001), which is designed to simultaneously adsorb and remove activated neutrophils, PNCs, and cytokines from the systemic circulation. In a previous study, NOA-001 treatment markedly improved pulmonary function in a rabbit model of hydrochloric acid/lipopolysaccharide-induced lung injury [8], although the study had limitations of limited assessment of lung edema formation and elucidation of the underlying mechanism.

In the present study, we hypothesized that selective removal of platelet-neutrophil complexes together with inflammatory cytokines by NOA-001 would suppress neutrophil-driven endothelial injury, thereby reducing pulmonary microvascular hyperpermeability, lung edema, and the severity of ARDS. This hypothesis was tested in a clinically relevant ovine model of smoke inhalation-induced ARDS, which has characteristics that closely resemble those of ARDS in humans.

## Materials and methods

### Animals

This study was approved by the Institutional Animal Care and Use Committee (IACUC) of the University of Texas Medical Branch (UTMB) (Galveston, TX, USA), and by the Animal Care and Use Committee of Toray Industries, Inc. The study was conducted in the translational intensive care unit at UTMB in compliance with the guidelines of *Animal Research: Reporting of *In Vivo* Experiments (ARRIVE)* [9], the National Institutes of Health, and the American Physiological Society for the Care and Use of Laboratory Animals [10].

### Surgical preparations

Adult female Merino sheep (30–46 kg) were surgically prepared with multiple catheters (pulmonary arterial [Swan-Ganz], left atrial, and right femoral arterial) placed to continuously monitor cardiopulmonary hemodynamics under anesthesia/analgesia and aseptic conditions [10]. Right carotid arterial and right jugular venous catheters were also placed for extracorporeal circulation. To measure lung lymph flow (pulmonary transvascular fluid flux [lung edema]), a thoracotomy in the fifth and seventh intercostal space was performed, and the efferent vessel of the caudal mediastinal lymph node was cannulated with Sillastic medical grade tubing (0.025-inch inner diameter, Dow Corning, Midland, MI) [11]. Long-acting (72 h) Buprenorphine SR™ (0.05 mg/kg, SR Veterinary Technologies, Windsor, CO) was used for pre- and post-surgical analgesia.

### Smoke inhalation injury

After a recovery period of 5–7 days, smoke inhalation injury was induced as previously described [10, 12]. Briefly, 48 breaths of cooled (< 40 °C) cotton smoke inhalation was conducted under deep anesthesia and analgesia (ketamine 20 mg/kg, isoflurane 1–5%, and Buprenorphine SR™ 0.1 mg/kg). Immediately after injury, anesthesia was discontinued and animals were allowed to waken while on mechanical ventilation (Hamilton-G5, Hamilton Medicals, Switzerland) in synchronized controlled mandatory ventilation (s-CMV) mode. Ventilator settings included a positive end-expiratory pressure (PEEP) of 5 cmH₂O, a tidal volume of 12 mL/kg, and a respiratory rate of 20 breaths per minute, which was adjusted as needed to maintain normocapnia (arterial carbon dioxide tension (PaCO₂) close to baseline). Sheep require a higher tidal volume than humans due to a larger anatomical dead space and ratio of dead space to tidal volume [13]. Animals received 100% oxygen for the first 3 h post-injury; thereafter, the fraction of inspired oxygen (FiO₂) was adjusted to maintain arterial oxygen tension (PaO₂) around 100 mmHg. All animals received fluid resuscitation with lactated Ringer’s solution at an initial rate of 2 mL/kg/h. The fluid rate was adjusted considering hematocrit changes to keep the baseline ± 3% range during the study. Animals were allowed free access to food, but not to water to control fluid intake. After smoke inhalation, animals with carboxyhemoglobin levels > 65% were randomly assigned to two experimental groups.

### Test column (NOA-001)

The NOA-001 blood purification column consists of polystyrene-based composition fibers reinforced with chemically modified polypropylene [14]. The fibers were treated with N-methylol-α-chloracetamide in a mixture of sulfuric acid and nitrobenzene on ice for 2 h in the presence of paraformaldehyde. They were subsequently reacted with tetraethylenepentamine and 4-chlorophenyl isocyanate in dimethyl sulfoxide (DMSO) for 3 h at 40 °C. After thorough washing with DMSO followed by methanol and pyrogen-free water, the 63 × 225 mm column was packed with 120 cm^3^ knitted fibers. The priming volume was approximately 162 mL, equivalent to the planned clinical application size. The column was filled with pyrogen-free saline, sterilized using gamma-ray irradiation (25,000 Gy), and stored at room temperature (< 30 °C) until use.

### Direct hemoadsorption

Extracorporeal circulation using the NOA-001 column was initiated 3 h after smoke inhalation injury, based on previous findings indicating significant changes in circulating neutrophils and their reactivity within 1–3 h post-injury [15], and to replicate conditions relevant to clinical use of extracorporeal circulation. The NOA-001 column was circulated with a flow rate of 100 mL/min through the arterio-venous circuit from 3 to 27 h post-injury. Immediately after initiating circulation, heparin was administered intravenously at 60 IU/kg as an anticoagulant and continuously infused to maintain an activated clotting time between 200 and 250 s. Animals were supported with mechanical ventilation and received fluid resuscitation. The control animals were circulated using the same blood circuit setup without the NOA-001 column.

### Measurements

Baseline endpoint variables and blood samples were collected from arterial and venous lines prior to injury (baseline). At 3 h post-injury, blood samples were collected again from arterial and venous sites, after which extracorporeal circulation was initiated. Subsequent blood samples were collected at 4, 6, 9, 12, 18, 24, and 27 h post-injury. Blood gas parameters were analyzed using a blood gas analyzer (RAPIDPoint 500; Siemens Healthcare, Erlangen, Germany) to assess pulmonary gas exchange. The oxygenation index (OI) was calculated as: OI = (FiO_2_ x Mean airway pressure) / PaO_2_. Blood samples for the inlet and outlet sides of the column at 4, 6, 9, 12, 18, 24 h after injury were measured using a hematology system (ADVIA 120; Siemens Inc., Malvern, PA, USA) to evaluate the neutrophil capture rate (NCR). NCR (%) was calculated as: NCR (%) = [(inlet neutrophil count—outlet neutrophils count) / inlet neutrophil count] × 100.

Pulmonary microvascular hyperpermeability to water, protein, and neutrophils was assessed by measuring the volume, protein content, and neutrophil counts in collected lung lymph. The pulmonary trans-endothelial protein flux index (PTPFI) was calculated as: PTPFI (mL/h) = (Lymph protein concentration x Lung lymph flow) / Plasma protein concentration. The pulmonary trans-endothelial neutrophil migration index (PTNMI) was calculated as: PTMNI = (Lung lymph neutrophil count x Lung lymph flow) / Blood neutrophil count. The lung injury score (LIS), which is used in clinical settings, was calculated using the P/F ratio, PEEP, lung compliance, and chest X-ray consolidations [16]. Cardiopulmonary hemodynamic parameters were continuously monitored for 27 h.

### Cytokine concentration

Bronchoalveolar lavage fluid samples were collected 24 h after hemoadsorption and diluted 15-fold with phosphate-buffered saline (PBS). Interleukin-6 (IL-6) levels were measured using an ELISA for ovine cytokines (ELISA Kit for IL-6, #SEA079ov, Cloud-Clone Corp., Katy, TX, USA).

### Flow cytometric analysis

Arterial blood samples were incubated with CD45-PE, CD11b-FITC, and CD62P-Alexa Fluor647 (Bio-Rad, Hercules, CA, USA). Red blood cells were lysed, and the remaining leukocytes and platelets were rinsed using buffer (PBS(-), 0.05% NaN_3_) and resuspended in formaldehyde-containing buffer. Cells were characterized by BD LSRFortessa (BD, Franklin Lakes, NJ, USA) and data were analyzed using FlowJo software (FlowJo, LLC, Ashland, OR, USA).

In CD45-positive granulocytes, levels of CD11b and CD62P were analyzed. Since an antibody specific to the activated conformation of CD11b is not available for ovine samples, CD11b expression intensity (high vs. low) was used as a surrogate marker of activation. Blood samples obtained prior to smoke inhalation were used as controls to determine CD11b^low^ and an isotype control-stained sample was used to determine the CD62P^–^ region. CD11b^low^ was determined as 90% of the control cells included, and CD62P^–^ as 95% of the isotype control-stained cells included. The absolute number of granulocytes was calculated by multiplying the percentage obtained from flow cytometry by the total granulocyte count from hematological analysis. The adsorption ratio (%) was calculated as: Adsorption Ratio = [(Inlet absolute cell count – Outlet absolute cell count) / Inlet absolute cell count] × 100. As most granulocytes were neutrophils, the granulocyte data were considered to primarily reflect neutrophil behavior.

### Necropsy

After 24 h of hemoadsorption, animals were anesthetized and subjected to chest X-ray. Then they were deeply anesthetized and euthanized by intravenous administration of xylazine (3 mg/kg), ketamine (40 mg/kg), and buprenorphine (0.01 mg/kg), in accordance with IACUC-approved protocols and American Veterinary Medical Association Guidelines for Euthanasia. Thoracic fluid was collected and measured, and lung tissue samples were collected, immediately snap-frozen, and stored at -80 °C. The bloodless lung wet-to-dry weight (W/D) ratio was determined using the lower half of the right lower lobe. Excised lung tissue was weighed and then dried to constant weight in an oven at 50 °C. Lung and trachea W/D ratios were also calculated [17].

### Statistical analysis

Data are expressed as the mean ± standard error of mean (SEM). Comparisons of groups were performed by mixed-model ANOVA with a Sidak post-hoc test or Mann–Whitney U test using Prism 6 (GraphPad Software, Inc. San Diego, CA, USA). A Wilcoxon signed-rank test was used to evaluate the change between 3- and 4-h time points. Cell count changes in flow cytometry were evaluated by Friedman test. p < 0.05 was considered statistically significant in all analyses.

## Results

### Injury severity and survival

Arterial blood carboxyhemoglobin levels following smoke inhalation injury did not differ significantly between the NOA-001 and control groups (76.8 ± 1.9% vs. 69.9 ± 2.6%, p = 0.07), indicating a similar degree of injury. All 11 animals (six in the NOA-001 group and five in the control group) were treated with extracorporeal circulation and survived for the full 27-h study period. There were no adverse events such as hypotension, anemia, bleeding or anticoagulation related problems in the NOA-001 group.

### Neutrophil capture and cytokine adsorption

**Fig. 1:**
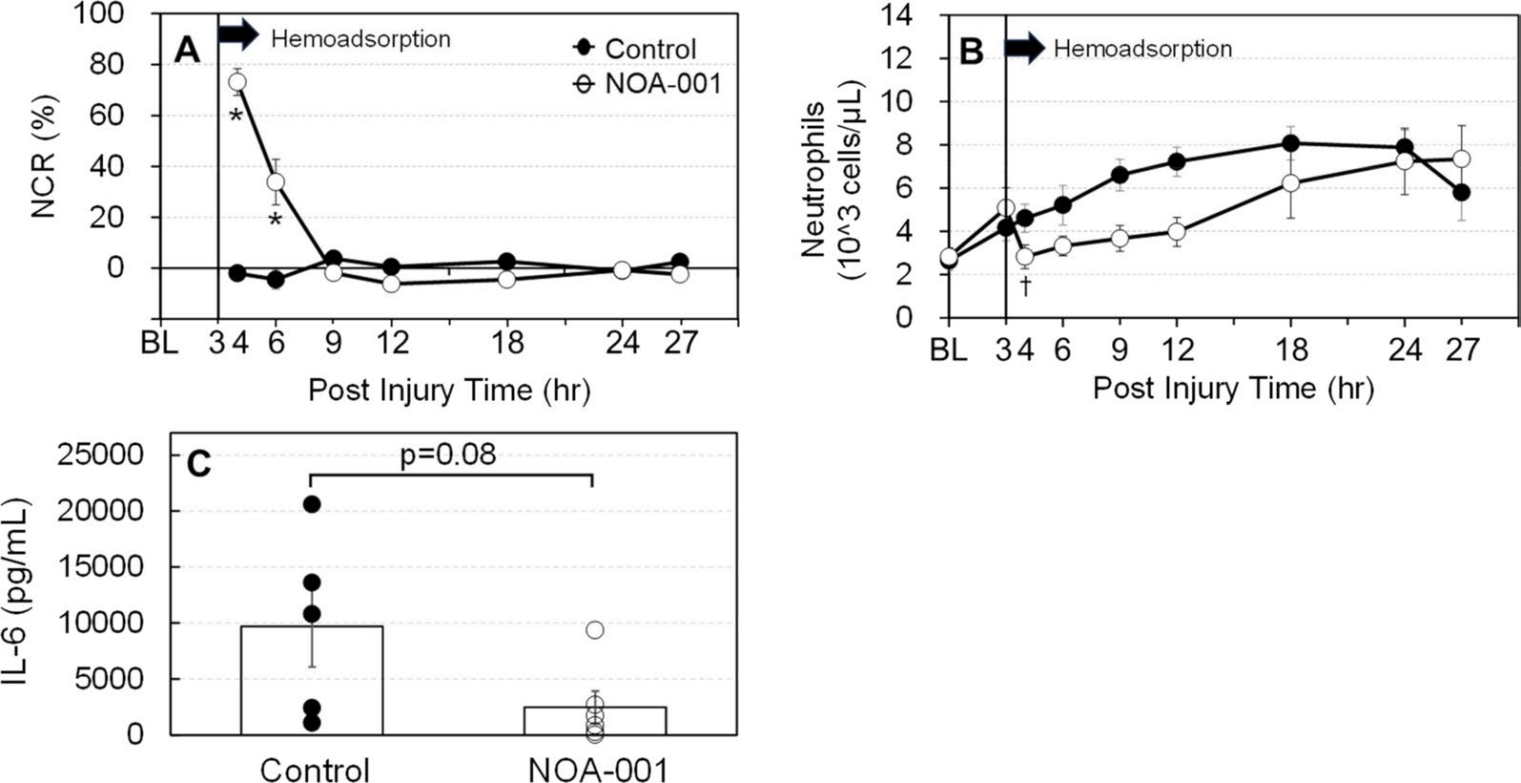
Changes in **A** systemic neutrophil capturing rate **B** systemic neutrophil counts and C concentration of IL-6 in Bronchoalveolar lavage fluid. * p < 0.05, mixed-model ANOVA with Sidak’s post-hoc test or Mann–Whitney U test (Mean ± SEM). ^†^p < 0.05, Wilcoxon signed-rank test was performed for analysis for the change degree between 3 and 4 h

At 4 and 6 h post-injury (1 and 3 h after the start of hemoadsorption), the NCR was significantly higher in the NOA-001 group compared to the control group (Fig. 1A). Systemic neutrophil counts in the NOA-001 group significantly decreased between 3 to 4 h and remained lower than those in the control group up to 12 h. From 12 to 24 h, neutrophil counts in the NOA-001 group gradually increased and reached levels similar to the control group by 24 h (Fig. 1B). The IL-6 level in bronchoalveolar lavage fluid at 27 h tended to be lower in the NOA-001 group compared to the control group (2,494 ± 1,427 vs. 9,697 ± 3,618 pg/mL, p = 0.08) (Fig. 1C).

### Flow cytometric assay

**Fig. 2:**
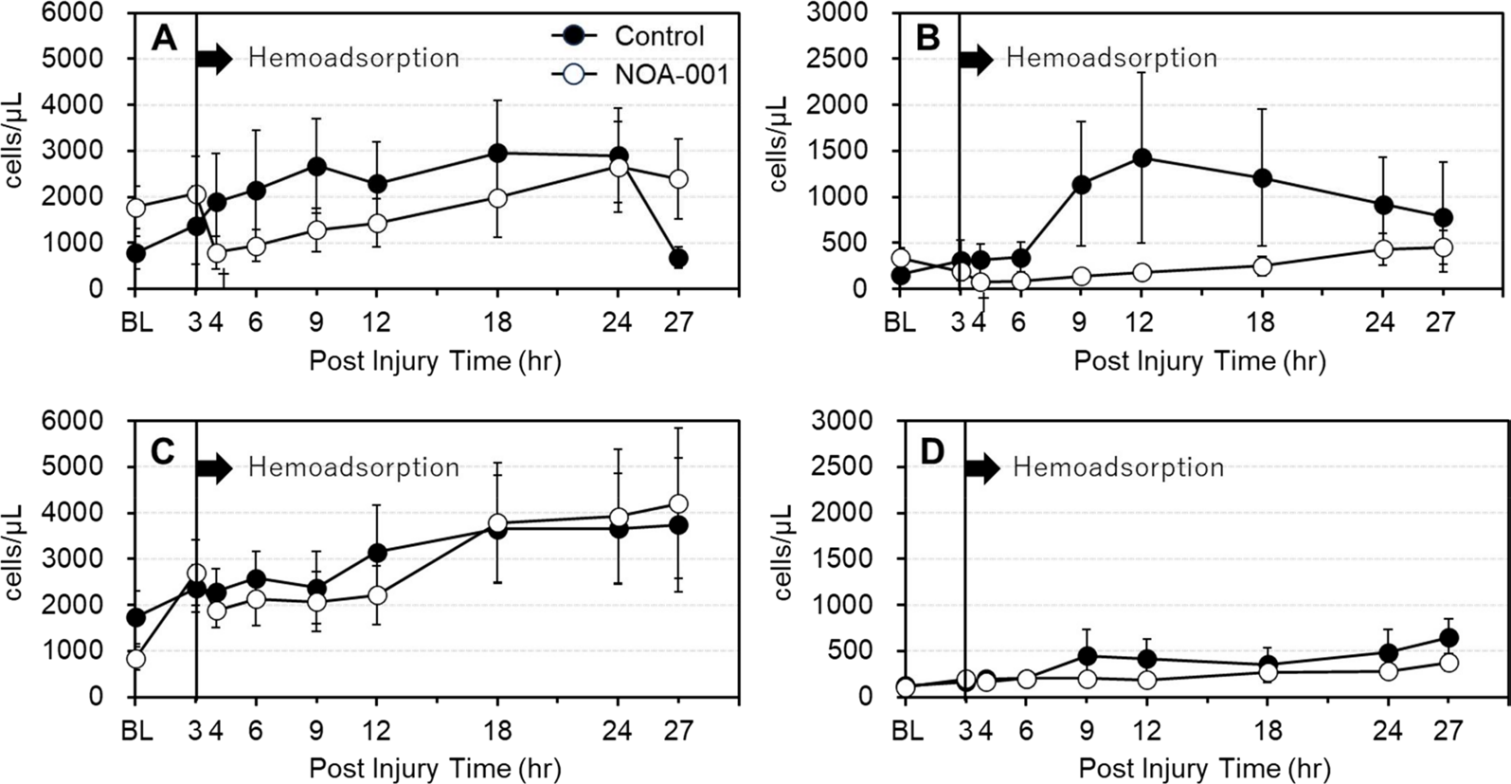
Changes in neutrophils, and platelet-neutrophil complex. **A** CD11b^low^ C62P^+^ **B** CD11b^high^ CD62p^+^, **C** CD11b^low^ CD62P-, **D** CD11b^high^ CD62p +. ^†^p < 0.05, Wilcoxon signed-rank test was performed for analysis for the change degree between 3 and 4 h. Friedman’s test was used for the analysis for cell increase in flow-cytometric assay

Neutrophils were identified by CD45 expression and activation was analyzed using the CD11b expression level. PNCs were identified by the presence of CD62P-positive cells among the neutrophils. In PNCs with low expression of CD11b (CD11b^low^) (Fig. 2A), cell counts in the NOA-001 group significantly decreased at 4 h, followed by a gradual increase. In contrast, the control group showed a continuous increase in these cells from baseline (0 h) to 24 h. In PNCs with high expression of CD11b (CD11b^high^), cell counts in the control group significantly increased at 12 h compared to baseline and reached 1500 cells/μL. In contrast, PNC/CD11b^high^ in the NOA-001 group significantly decreased at 4 h, and did not increase over the study period except at 27 h (Fig. 2B). Changes in CD11b^low^/CD62P^−^ neutrophils (Fig. 2C) were similar in the two groups. A small number of CD11b^high^/CD62P^–^ neutrophils were detected, with an increase from 100 cells/µL at baseline to 400–650 cells/µL at 27 h in both groups; however, these changes were not significant (Fig. 2D).

### Oxygenation index and lung injury score

**Fig. 3:**
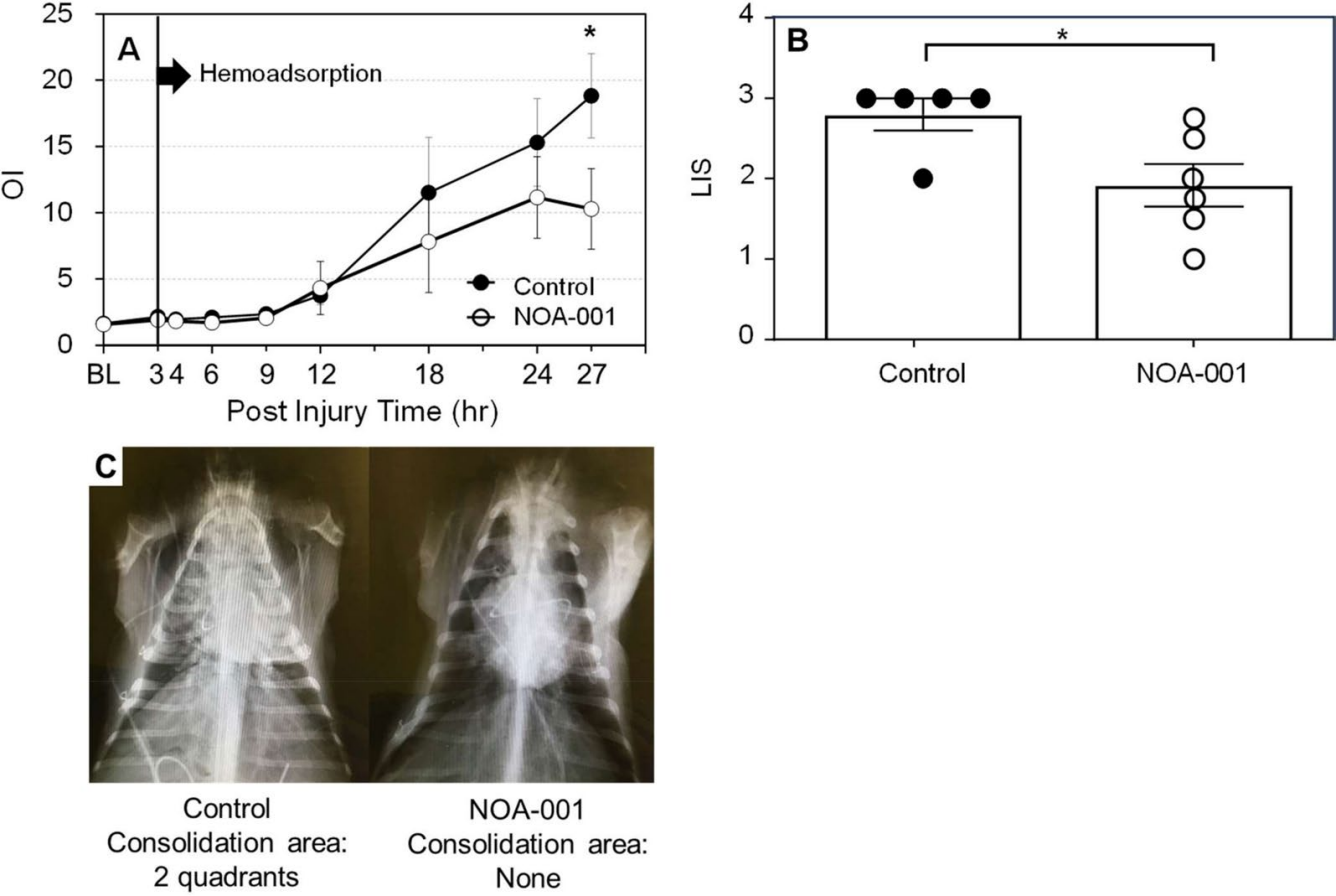
**A** Changes in oxygenation index (OI). **B** Lung injury Score (LIS) at 27 h after smoke injury. **C** Consolidation of chest X-ray at 27 h. *p < 0.05, Mixed-model ANOVA with Sidax’s post-hoc test or Mann–Whitney U test (Mean ± SEM)

OI increased in the NOA-001 and control groups after 9 h, indicating deteriorated pulmonary function. However, OI in the NOA-001 group peaked at 24 h and was significantly lower than OI in the control group at 27 h (10.3 ± 3.0 vs. 18.8 ± 3.2, p < 0.05) (Fig. 3A). LIS at 27 h was also significantly lower in the NOA-001 group (1.9 ± 0.3 vs. 2.8 ± 0.2, p < 0.05) (Fig. 3B). X-ray images (Fig. 3C) showed lung consolidation in two quadrants in the control group, but no consolidation in the NOA-001 group.

### Lung lymph flow measurements

**Fig. 4:**
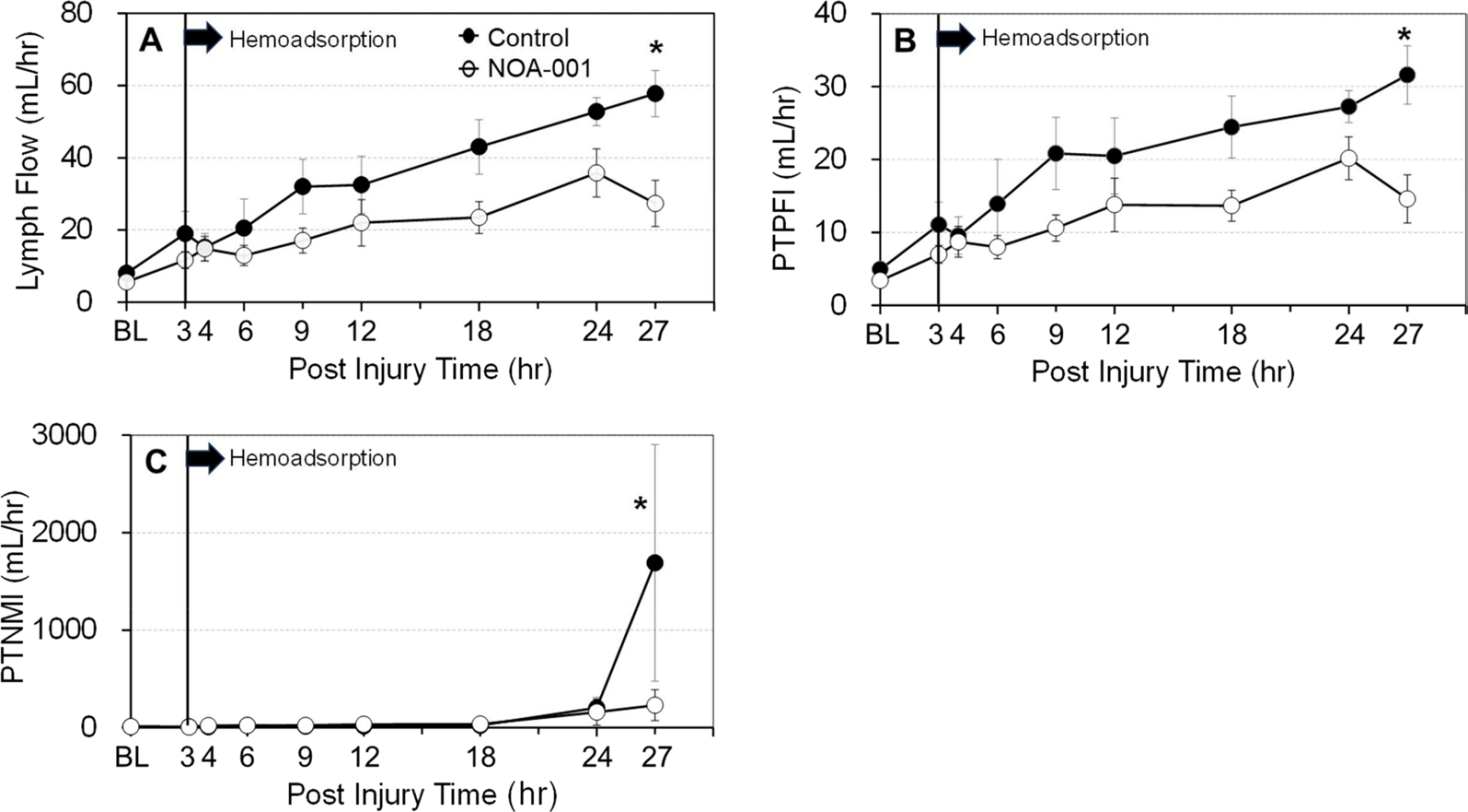
Changes in **A** Ltymph flow, **B** Pulmonary transendothelial protein flux index (PTPFI), and **C** Pulmonary transendothelial neutrophil migration index (PTNMI). *p < 0.05, Mixed-model ANOVA with Sidax’s post-hoc test

Lung lymph flow, an indicator of pulmonary transvascular fluid flux, gradually increased in both groups (Fig. 4A), but tended to be lower in the NOA-001 group throughout the study period, and was significantly reduced at 27 h compared to the control group (27.3 ± 6.4 vs. 57.8 ± 6.4 mL/h p < 0.05). A similar trend was observed for PTPFI, which was significantly lower in the NOA-001 group (14.6 ± 3.3 vs 31.6 ± 4.0 mL/h, p < 0.05). The increase in PTNMI remained low in both groups until 24 h; however, PTNMI was significantly elevated in the control group compared to the NOA-001 group at 27 h (230.7 ± 157.2 vs. 1889.3 ± 1692.7 mL/h, p < 0.05) (Fig. 4C).

### Organ wet-to-dry weight ratio and pleural effusion

**Fig. 5:**
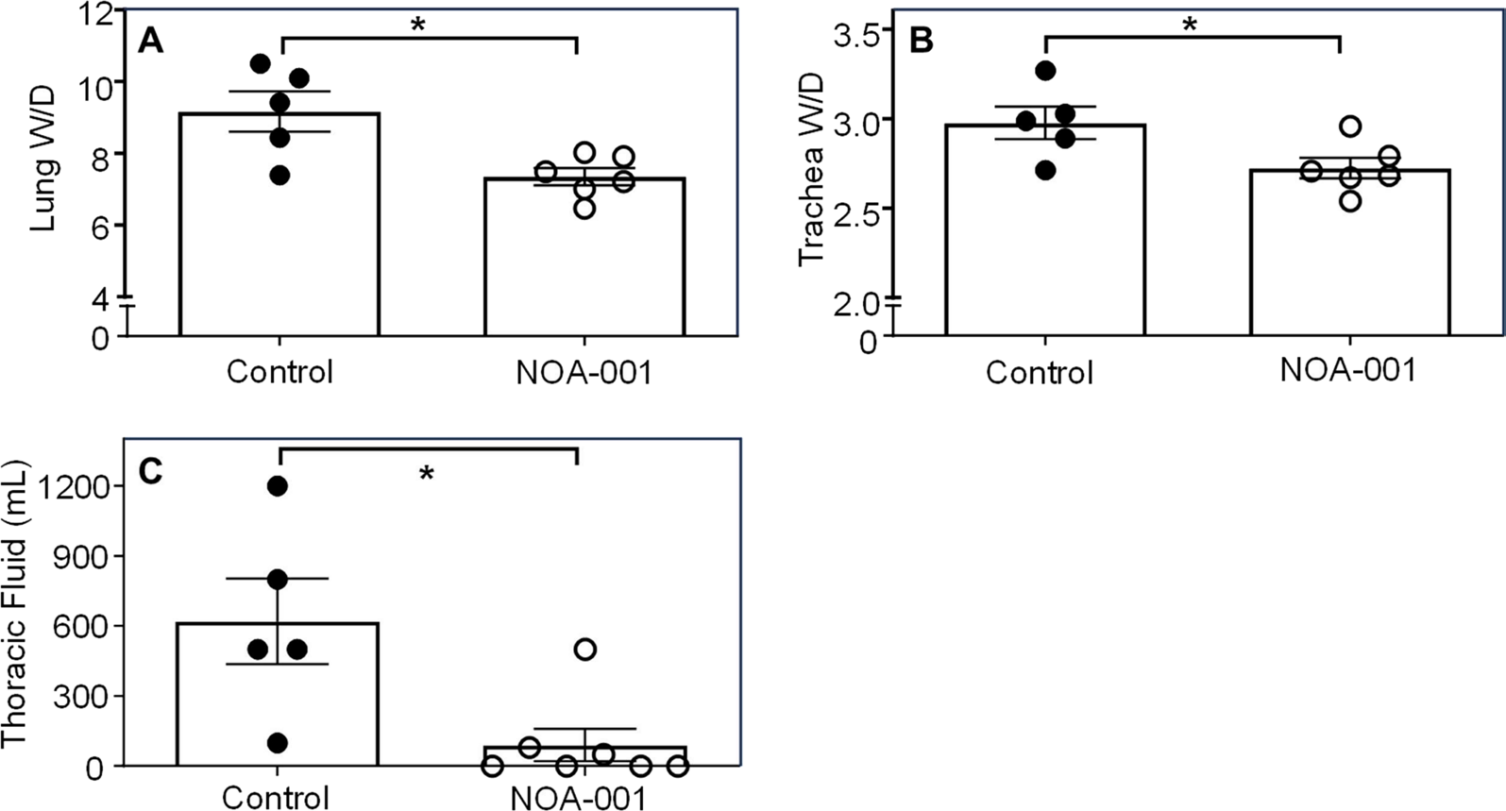
**A** Lung bloodless wet-to-dry weight ratio, **B** Trachea bloodless wet-to-dry weight ratio, and C Thoracic fluid after 27 h treatment. *p < 0.05, Mann–Whitney U test (Mean ± SEM)

The lung bloodless W/D ratio was significantly lower in the NOA-001 group compared to the control group (7.4 ± 0.2 vs. 9.2 ± 0.6, p < 0.05) (Fig. 5A). Of note, the W/D ratio in the NOA-001 group was higher than the normal range determined in a sham group (3.74 ± 0.35) in a previous study [18]. The tracheal W/D ratio was also significantly lower in the NOA-001 group compared to the control group (2.7 ± 0.1 vs. 3.0 ± 0.1, p < 0.05) (Fig. 5B). The NOA-001 group also had significantly less pleural effusion at necropsy (90 ± 69 vs. 620 ± 183 mL, p < 0.05) (Fig. 5C).

### Hemodynamics, airway pressures, and biochemical parameters

Body temperature and cardiac output increased in the control group and differences with the NOA-001 group were significant at 18 and 24 h (Table 1). Peak and plateau airway pressures and blood glucose and lactate levels did not differ significantly between the two groups (Table 1).

**Table 1:** Cardiopulmonary hemodynamics and biochemical variables from baseline to 27 h

Parameter	Group/Time	BL	3 h	6 h	12 h	18 h	24 h	27 h
BT (°C)	Control	39.2 ± 0.1	39.6 ± 0.1	39.7 ± 0.3	39.4 ± 0.2	39.6 ± 0.3*	39.7 ± 0.3*	39.6 ± 0.2
	Treatment	39.1 ± 0.1	39.7 ± 0.1	39.4 ± 0.1	39.5 ± 0.2	39.5 ± 0.2*	39.6 ± 0.2*	39.5 ± 0.2
HR (beats/min)	Control	88 ± 4	95 ± 5	105 ± 6	110 ± 8	122 ± 8	116 ± 8	123 ± 10
	Treatment	91 ± 4	100 ± 3	103 ± 5	114 ± 9	117 ± 5	128 ± 9	128 ± 10
MAP (mmHg)	Control	99 ± 2	112 ± 2	112 ± 3	107 ± 2	105 ± 3	109 ± 3	107 ± 3
	Treatment	99 ± 3	110 ± 3	114 ± 3	112 ± 3	110 ± 2	109 ± 3	109 ± 3
CI (mL/min/m^2^)	Control	5.9 ± 0.2	6.2 ± 0.3	6.3 ± 0.5	5.4 ± 0.4	5.9 ± 0.5*	5.3 ± 0.4*	5.8 ± 0.5
	Treatment	5.9 ± 0.3	6.4 ± 0.3	6.0 ± 0.3	5.8 ± 0.2	5.7 ± 0.3*	5.9 ± 0.4*	6.5 ± 0.5
PAP (cmH_2_O)	Control	18 ± 1	18 ± 1	22 ± 3	22 ± 1	29 ± 2	33 ± 2	38 ± 2
	Treatment	18 ± 1	17 ± 1	16 ± 1	23 ± 2	26 ± 2	36 ± 3	35 ± 3
PLAP (cmH_2_O)	Control	17 ± 1	17 ± 1	18 ± 2	20 ± 1	27 ± 2	31 ± 2	35 ± 2
	Treatment	17 ± 1	16 ± 1	15 ± 1	23 ± 2	25 ± 2	33 ± 3	33 ± 3
Glucose (mg/dL)	Control	68 ± 3	84 ± 5	78 ± 6	81 ± 5	77 ± 5	79 ± 6	84 ± 9
	Treatment	58 ± 2	80 ± 4	73 ± 3	73 ± 4	75 ± 5	83 ± 7	80 ± 9
Lactate (mmol/L)	Control	0.5 ± 0.1	1.3 ± 0.1	1.6 ± 0.2	1.5 ± 0.2	1.0 ± 0.1	1.2 ± 0.2	1.6 ± 0.3
	Treatment	0.7 ± 0.1	1.8 ± 0.3	1.7 ± 0.3	1.5 ± 0.2	1.5 ± 0.3	1.2 ± 0.1	1.5 ± 0.3

Data are expressed as mean ± SEM. *: p < 0.05 for treatment vs. control group

BL: baseline, BT: body temperature, HR: heart rate, MAP: mean arterial pressure, CI: cardiac Index, PAP: peak airway pressure, PLAP: plateau airway pressure

## Discussion

In this clinically relevant ovine model of smoke inhalation-induced ARDS, we found that simultaneous extracorporeal removal of cytokines and PNCs using a novel hemoadsorption column NOA-001 significantly attenuated lung injury. NOA-001 treatment resulted in preferential removal of circulating PNCs, particularly CD62P-positive neutrophils, and was associated with reduced pulmonary microvascular hyperpermeability, as evidenced by lower lung lymph flow, decreased trans-endothelial protein and neutrophil flux, reduced lung and tracheal edema, and diminished pleural effusion. These physiological improvements translated into clinically meaningful outcomes, including improved oxygenation index and lower lung injury scores, without adverse hemodynamic effects. Collectively, these findings indicate that targeting both inflammatory mediators and immuno-thrombotic cell complexes can effectively mitigate ARDS severity.

Adsorption of PNCs by NOA-001 was achieved using their intrinsically high phagocytic activity. Controlled surface roughness was engineered on the fibrous adsorbent to mimic bacterial size [8], thereby inducing active adhesion by leukocytes with robust phagocytic activity, such as PNCs, through their inherent foreign-body removal mechanisms. In contrast, non-activated neutrophils with low phagocytic activity have minimal adhesion to the fibers, enabling selective capture. Cytokine adsorption was facilitated by introducing specific chemical structures [14] onto the fibers, giving selective affinity (adsorption rates: CD11b + /CD62P + cells = 60.9%, CD11b + /CD62P- cells = 9.9%, and CD11b- cells = -5.5% [8]) through molecular interactions. Also, the adsorption fiber is negative for hemolysis and does not adsorb blood components such as albumin, immunoglobulins, complement, or lipids, but can adsorb certain pharmaceuticals, including vancomycin [14].

PNCs participate in the inflammatory process and are implicated in organ failure in patients with ARDS [19]. Increased levels of PNCs have also been observed in patients with COVID-19 [20]. There are at least two subtypes of PNCs (CD11b^low^ and CD11b^high^). In the control group, increases in lung lymph flow and PTPFI started at 3 h post-injury, and an increase in PNC/CD11b^low^ also occurred at this time point. An increase in OI was observed at 9 h post-injury, aligning with the increase in PNC/CD11b^high^. Taken together, these temporal associations suggest that PNCs contribute to development of ARDS and that their removal or deactivation may be a promising therapeutic option for ARDS.

Flow cytometric analysis showed that CD62P-positive neutrophils were the main cell population to be reduced by NOA-001 hemoadsorption, indicating effective removal of PNCs. This finding is consistent with in vitro studies showing that NOA-001 eliminates PNCs in lipopolysaccharide (LPS)-activated human blood samples. Moreover, NOA-001 treatment produced a major reduction in PNC/CD11b^low^ cells at 4 h, and the increase of PNC/CD11b^high^ until 12 h was slight. The time courses of PNC transition and pathological progression with NOA-001 treatment suggests that increased hyperpermeability of water and proteins in lung vasculature was suppressed by removal of PNC/CD11b^low^ in the early phase, followed by a low level of more invasive PNC/CD11b^high^, thus contributing to suppression of deterioration of oxygenation ability.

Although neutrophils contribute to tissue injury in inflammatory states, they also serve critical roles in host defense, particularly at infection sites. Formation of PNCs is also associated with development of NETs as a host defense system against microorganisms by regulating the innate immune response [5]. Therefore, any strategy aiming to eliminate neutrophils or PNCs must selectively target the subpopulations associated with tissue injury while preserving those involved in antimicrobial defense. Our data support this selectivity, since CD11b^low^/CD62P^−^ neutrophils were only removed slightly by NOA-001 treatment, with no significant difference with the control. This suggests that neutrophils that are not activated or complexed with platelets were not removed by the NOA-001 column and remained in the systemic circulation. Furthermore, NOA-001 adsorbs only systemically circulating cells and does not affect immune cells engaged at local infection sites. This site-specific effect is a potential advantage of extracorporeal hemoadsorption over systemic therapies such as corticosteroids, which broadly suppress leukocyte activity and carry a risk of immunosuppression [21]. Hemoadsorption treatment can also be halted if immunosuppression becomes a concern.

Sekiya et al. showed the effectiveness of NOA-001 in a LPS/acid inhalation rabbit model of lung injury using 8-h hemoadsorption treatment [8]. Although effective, the study was limited by the short observation time and restricted blood sampling. The current ovine study used a 24-h hemoadsorption protocol, enabling more comprehensive evaluation of the mechanism of NOA-001 and efficacy. Importantly, NOA-001 treatment was associated with a reduction in PNCs/CD11^high^ levels by 12 h post-injury compared to the control group, and also reduced neutrophil migration into lung lymph at 24 h after treatment. These findings suggest inhibition of trans-endothelial neutrophil migration across the kung endothelium.

The ovine model is still limited by a relatively short-term (27-h) duration and its inability to provide a mechanistic link between reduction of PNCs in the systemic circulation and lung vascular hyperpermeability. However, the model recapitulated key clinical features of ARDS, supporting its value as a preclinical translational model. Use of NOA-001 was not associated with adverse cardiopulmonary hemodynamic effects.

## Conclusions

The novel hemoadsorption column NOA-001, designed for simultaneous removal of cytokines and activated neutrophils (including PNCs), attenuated the severity of smoke inhalation-induced ARDS in sheep. This effect involved reduction of pulmonary microvascular hyperpermeability and edema, improved pulmonary gas exchange, and reduction of LIS, mainly via elimination of pro-inflammatory factors such as PNCs from the systemic circulation. Importantly, NOA-001 was safe to use over a 24-h period. Further studies are warranted to confirm the safety and efficacy of long-term NOA-001 use for various types of ARDS in the ICU population in clinical settings.

## Data Availability

Datasets used in the study are available from the corresponding author on reasonable request.

## Abbreviations

ARDS: Acute respiratory distress syndrome
PNCs: Platelet-neutrophil complexes
IACUC: Institutional Animal Care and Use Committee
UTMB: University of Texas Medical Branch
NETs: Neutrophil extracellular traps
s-CMV: Synchronized controlled mandatory ventilation
PEEP: Positive end-expiratory pressure
FiO₂: Fraction of inspired oxygen
PaO₂: Arterial oxygen tension
DMSO: Dimethyl sulfoxide
OI: Oxygenation index
NCR: Neutrophil capture rate
PTPFI: Pulmonary trans-endothelial protein flux index
PTNMI: Pulmonary trans-endothelial neutrophil migration index
LIS: Lung injury score
PBS: Phosphate-buffered saline
IL-6: Interleukine-6
W/D: Wet-to-dry weight ratio
LPS: Lipopolysaccharide
